# Pattern of Lung Involvement in Predicting Severity and Sequelae in Patients With COVID-19

**DOI:** 10.7759/cureus.32973

**Published:** 2022-12-26

**Authors:** Jennie Santhanam, Ankush Agarwal S, Sarah Mammen, Arun K, Aishwarya V Athani, Subramaniyan K, Meenakshi Sundari, Hussain Ibrahim, Uthaya Nila

**Affiliations:** 1 General Medicine, Sri Ramaswamy Memorial (SRM) Medical College Hospital and Research Centre, Chengalpattu, IND; 2 Radiology, Sri Ramaswamy Memorial (SRM) Medical College Hospital and Research Centre, Chengalpattu, IND; 3 Internal Medicine, Sri Ramaswamy Memorial (SRM) Medical College Hospital and Research Centre, Chengalpattu, IND

**Keywords:** lung findings in covid, world pandemic, ggo (ground glass opacities), hrct chest (high resolution computed tomography), covid 19

## Abstract

Background

During the COVID pandemic, high-resolution CT scan has played a pivotal role in detecting lung involvement and severity based on the segments of the lung involved. The pattern of involvement was not considered, and our aim is to observe the pattern of lung involvement in predicting severity and guiding management protocol in patients with COVID-19.

Methodology

It was a prospective observational study conducted with 151 patients admitted with COVID-19 with a positive reverse transcriptase polymerase chain reaction test (RT-PCR) in a single tertiary care hospital in south India. Patients with pre-existing lung pathologies were excluded from the study. Eligible patients were then divided into mild, moderate, and severe categories based on Indian Council of Medical Research (ICMR) guidelines, and high-resolution computed tomography (HRCT) chest was done, findings of which were then categorized based on lung involvement; into ground glass opacities (GGO), interstitial involvement and mixture of both. These were then analyzed to determine their importance with respect to the duration of stay and severity of the disease.

Results

The data collected was analyzed by IBM SPSS software version 23.0 (IBM Corp., Armonk, NY, USA). The study population included 114 males (75.5%) and 37 females (24.5%). HRCT chest was done which showed 62.3% of patients had GGO, 14.6% had interstitial lung involvement, 18.5% had a mixture of both and 4.6% had normal lung findings. These findings, when compared to clinical categories of severity, showed a significant co-relation between pattern of involvement of the lung and the severity of the disease. It also showed significant co-relation with the duration of stay.

Conclusion

HRCT chest has proven to be useful in the determination of patient’s severity and can guide with management. We suggest earlier initiation of steroids and anticoagulants in patients with interstitial involvement even for the patients not on oxygen therapy yet. It can be used as a triage modality for screening due to the advantage of presenting with immediate results as opposed to RT-PCR which might take hours and can delay treatment which can prevent worsening.

## Introduction

The novel coronavirus (COVID-19) was first encountered at the end of 2019 in the city of Wuhan in Hubei province of China. Since its first occurrence, we have encountered multiple waves and various mutant strains which have taken a toll on the physical, emotional, financial, and economic well-being of various nations and their people. COVID-19 was declared a pandemic by the World health organization (WHO) in March 2020 [1]. Its propensity to cause pneumonia and resulting death has perplexed scientists all around the world and studies were conducted to determine the severity of the disease for more aggressive treatment protocols to be initiated.

High-resolution computed tomography (HRCT) chest was used for detecting lung involvement in covid patients and as a screening tool for asymptomatic patients planned for elective surgeries during the pandemic [2]. It has helped determine the severity of the infection based on the segments involved but not the pattern of involvement.

Lung findings in covid patients could involve pulmonary arterioles or venules, depending on which there may be tissue damage visible in HRCT. A blockage of pulmonary arterioles would cause the formation of ground glass opacities (GGOs) without necessarily causing septal thickening, whereas a blockage of pulmonary venules would cause a predominance of GGOs with septal thickening [2].

In this study, we wished to assess the prognostic implication of HRCT chest in determining the severity of COVID-19 based on the pattern of lung involvement. We also aimed to determine its relation to the clinical categorization of severity and duration of stay in the hospital.

## Materials and methods

A prospective study was conducted from December 2020 to May 2021 at Sri Ramaswamy Memorial (SRM) Medical College Hospital and Research Centre in Chengalpattu district of Tamil Nadu, India. Institutional Ethics Committee approval was obtained with approval number 2379/IEC/2020. Patients of all age groups with positive COVID-19 reverse transcriptase-polymerase chain reaction test (RT-PCR) were included in the study. Patients were selected after excluding pre-existing lung diseases such as fibrosis, bronchiectasis and lung carcinoma. Patients with active tuberculosis (TB) or on treatment for the same were also excluded from the study. Patients with other causes of sepsis may hinder clinical evaluation and laboratory findings and hence were also excluded. A total of 151 patients fulfilling the inclusion and exclusion criteria were included in the study after an informed consent.

All patients underwent clinical evaluation with detailed history, vitals, and examination. Their oxygen saturation at presentation and d-dimer levels were recorded. Based on Indian Council of Medical Research (ICMR) guidelines, patients were categorized into mild (patients who exhibit any of the several coronavirus (COVID-19) symptoms, e.g., fever, cough, sore throat, malaise, headache, muscle pain, without shortness of breath, dyspnoea, or abnormal chest X-ray), moderate (patients with lower respiratory disease as determined by clinical examination or imaging and an oxygen saturation level (SpO2) of greater than 94% in room air at sea level) and severe (respiratory rate greater than 30 breaths per minute, room air oxygen saturation (SpO2) less than 94%, arterial partial pressure of oxygen to fraction of inspired oxygen (PaO2/FiO2) less than 300 mmHg, or lung infiltrates greater than 50%).

Patients were subjected to an HRCT chest and findings were recorded. Operational definitions for the different patterns involved include alveolar, where there is a presence of GGOs without septal thickening; interstitial, which represents predominantly septal thickening along with small amounts of GGO; and mixed pattern (50/50) representing the equal presence of GGO and septal thickening along with other features such as subpleural atelectasis. Patients were followed up during the stay in the hospital and after six weeks, their severity with the duration of stay in the hospital was noted. For the purpose of the study, long duration represents more than three weeks of hospitalization, moderate represents 1-3 weeks duration and short represents less than one week duration of stay in hospital. Patients were also subjected to a repeat CT scan after six weeks of initial presentation to determine the progression or regression of lung involvement.

The machine used was a multidetector computed tomography (CT) with scanning parameters same as the one recommended for the thorax by the manufacturer. The images were analyzed by a single experienced radiologist.

## Results

The study was conducted with 151 patients with laboratory (RT-PCR) confirmed cases of COVID-19 with a mean age of 47 years with the majority being males (n = 114, 75.5%). Among these 151 patients, 85 (56.3%) had mild, 43 (28.5%) had moderate and 23 (15.2%) had severe COVID-19 infection according to the ICMR guidelines (Table 1).

**Table 1:** Distribution of study population based on the clinical severity of the disease

Severity of COVID-19 Infection	Frequency (n)	Percentage of Study Population
Mild	85	56.3%
Moderate	43	28.5%
Severe	23	15.2%

Patients were also followed up to determine the duration of stay in the hospital, 67 (44.4%) patients had a long duration of stay, 16 (10.6%) had a moderate and 68 (45%) had a short duration of stay (Table 2).

**Table 2:** Distribution of study population based on the duration of stay in the hospital

Duration of stay in hospital	Frequency (n)	Percentage of the study population
Long	67	44.4%
Moderate	16	10.6%
Short	68	45%

HRCT chest was done for all patients and the predominant finding noted in the patient population was an alveolar pattern with ground glass opacities (GGO) present in 94 (62.3%) patients. An interstitial pattern was noted in 22 (14.6%) patients and mixture of both was noted in 28 (18.5%) patients. Seven (4.6%) patients had normal HRCT chest findings (Table 3).

**Table 3:** Distribution of study population based on pattern of lung involvement GGO: Ground glass opacities

Pattern of lung involvement	Frequency (n)	Percentage of the study population
Normal	7	4.6%
Interstitial	22	14.6%
Alveolar (GGO)	94	62.3%
Mixed	28	18.5%

After data analysis, the study revealed that a significant (chi-square test, p <0.05) number of patients with severe infection had mixed pattern of lung involvement. Patients with mild infection had more of alveolar and the ones with moderate infection had interstitial pattern predominantly (Table 4).

**Table 4:** Pattern of lung involvement when compared to clinical severity GGO: Ground glass opacities

			Pattern				Chi-Square Test
			GGO	Interstitial	Mixed	Normal	
Severity	Mild	Count	70	4	4	7	
		%	74.5%	18.2%	14.3%	100.0%	P-value- 0.0005
	Moderate	Count	22	10	11	0	
		%	23.4%	45.5%	39.3%	0.0%	
	Severe	Count	2	8	13	0	
		%	2.1%	36.4%	46.4%	0.0%	
Total		Count	94	22	28	7	
		%	100.0%	100.0%	100.0%	100.0%	

This study also showed a significant co-relation between the duration of stay and the pattern of lung involvement (Table 5). Patients with predominantly alveolar pattern had a shorter duration of stay whereas most patients with mixed and interstitial pattern had a long duration of stay in the hospital.

**Table 5:** Co-relation of the duration of stay in the hospital with the pattern of lung involvement GGO: Ground glass opacities

			Pattern				Chi-Square Test
			GGO	Interstitial	Mixed	Normal	
Duration of stay	Long	Count	27	17	23	0	P-value- 0.0005
		%	28.7%	77.3%	82.1%	0.0%	
	Moderate	Count	11	3	2	0	
		%	11.7%	13.6%	7.1%	0.0%	
	Short	Count	56	2	3	7	
		%	59.6%	9.1%	10.7%	100.0%	
Total		Count	94	22	28	7	
		%	100.0%	100.0%	100.0%	100.0%	

On follow-up after six weeks of initial presentation, seven patients of the mixed pattern had succumbed to death. A total of 102 patients were subjected to repeat CT scan, 34 patients of the mixed and interstitial pattern of lung involvement had developed fibrosis resulting in long-term requirement of chest physiotherapy and decrease in residual lung capacity as witnessed with spirometry test. Sixty-eight patients of the alveolar group had complete resolution of lung findings. Rest of the patients were lost to follow-up. Statistical co-relation could not be done, and observational findings were noted.

## Discussion

The World Health Organization (WHO) designated coronavirus disease 2019 (COVID-19) a pandemic on March 11th, 2020, mostly because of the disease's rapid and extensive spread [1]. This virus elicits a hyperimmune response from the host which is termed as ‘cytokine storm’ resulting in extensive tissue damage and coagulopathy [2]. The etiologic agent of COVID-19 was isolated and identified as a novel coronavirus, initially designated as 2019-nCoV [3]. The virus was later sequenced, and the International Committee for Taxonomy of Viruses named it severe acute respiratory syndrome coronavirus-2 (SARS-CoV-2) [4].

COVID-19 is an enveloped virus made up of icosahedral, symmetrical particles with membrane-mounted spikes. They contain a huge, 26-32 kb single-stranded positive-sense, non-segmented RNA genome [5]. It attaches to the host cell by binding to the angiotensin-converting enzyme receptor 2 (ACE 2) with its virion spike protein [6]. It has evolved over time to form a few mutations which were later termed as “variant of concern.” WHO gave designated labels in the form of Greek alphabets such as alpha, beta, gamma, and delta for these mutations [7]. Globally over 500 million confirmed cases of COVID-19 have been reported and many more were unreported due to minor or no symptoms.

The name "microCLOTS" (microvascular COVID-19 lung vessels obstructive thromboinflammatory syndrome) was developed by Italian researchers for the abnormalities seen in the lung as a result of an inflammatory reaction and pulmonary thrombosis in the microvasculature [8]. It signified the importance of anticoagulation in covid patients and gave a pathophysiological process of lung involvement. It was noted that the lung changes occurred five to seven days after the onset of symptoms and CT scan done earlier may result in false negative reports [2]. Patients presenting with symptoms of hypoxia may require HRCT chest at the first visit to determine the extent of lung involvement and help with treatment protocols.

**Figure 1:**
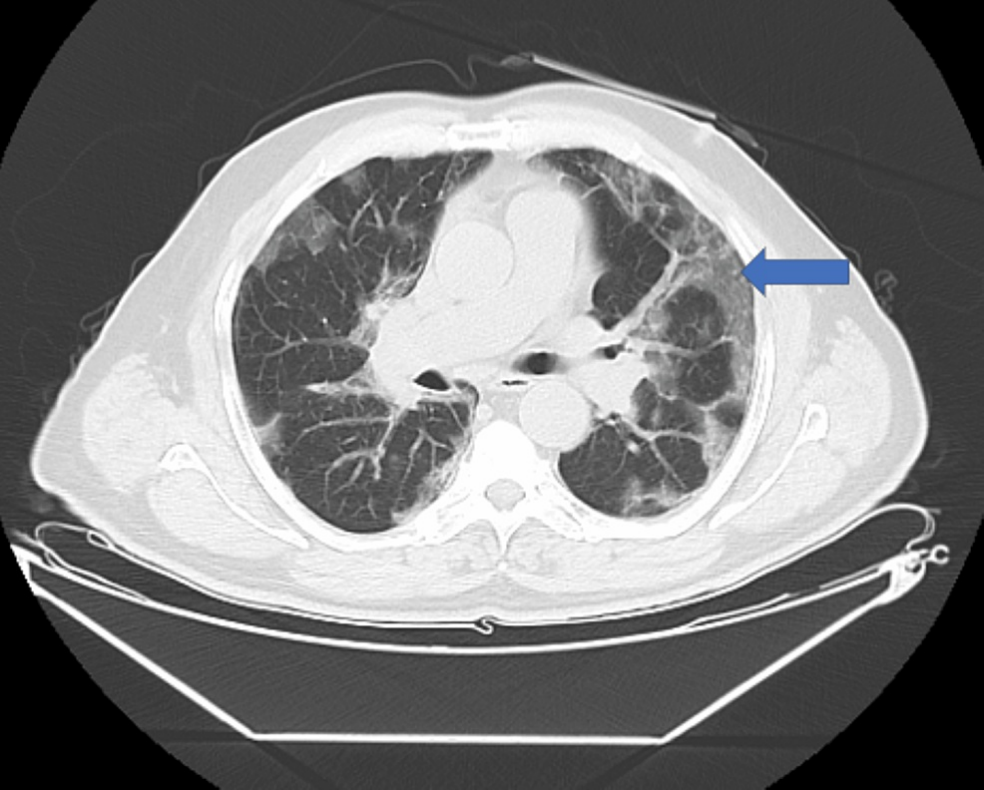
Ground glass opacities (blue arrow)

**Figure 2:**
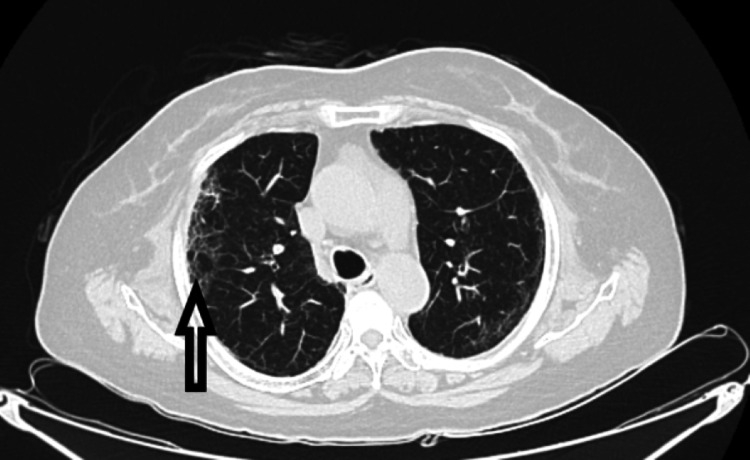
Diffuse reticular opacities with interlobular septal thickening (black arrow)

HRCT scan can present two different patterns of lung involvement, alveolar and interstitial [2]. Pulmonary arteritis along with the involvement of airspace due to the expression of angiotensin-converting enzyme receptors (ACE) in the acini results in ground glass opacities without septal thickening (Figure 1) and with progression into the lobular parenchyma causes septal thickening due to involvement of pulmonary veins (Figure 2) [2].

**Figure 3:**
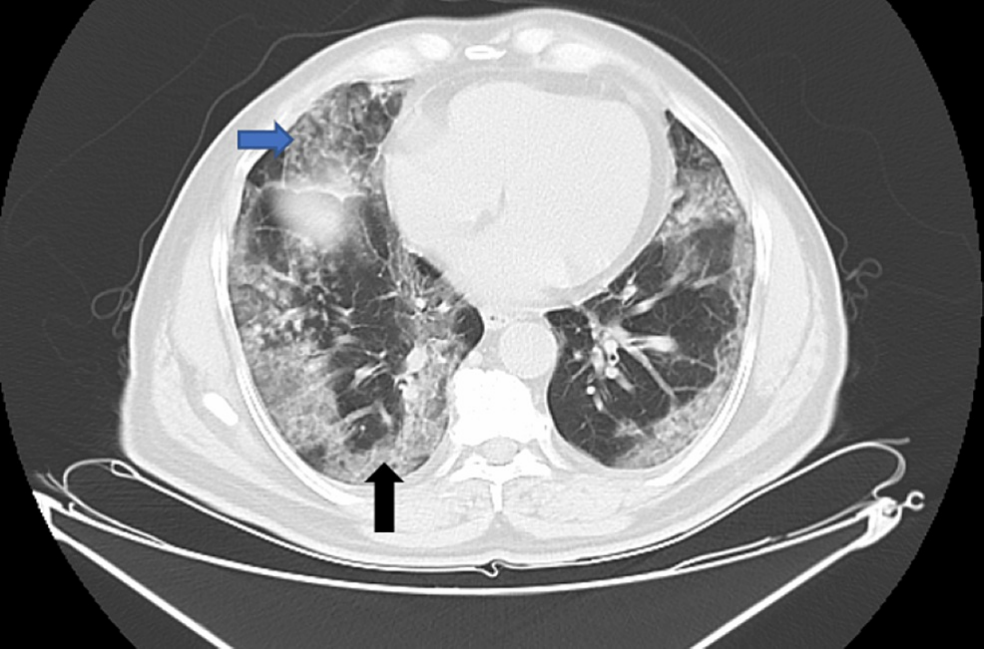
Mixed pattern of lung involvement (black arrow represents GGO, blue arrow points to septal thickening) GGO: Ground glass opacities

Some patients may present with a mixed pattern of lung involvement (Figure 3). This is supported by Tian et al. who reported lung histopathology of two patients who underwent lung lobectomy and retrospectively had infection at the time of surgery, authors reported vascular congestion combined with inflammatory fibrinoid materials [9]. Another study by Zhang et al. noted intravascular coagulopathy with vascular dysfunction by doing a transthoracic needle lung biopsy. The findings histopathologically were characterized by congestion of capillaries, pneumocyte necrosis, interstitial edema, hyperplasia of pneumocytes, atypia and platelet-fibrin thrombi [10].

The CT severity score indicates the segmental and lobar lung involvement caused by COVID-19 [11], which by itself is unable to indicate which subsets will get worse as the illness progresses. The pattern of lung involvement is not taken into account. The significance of the same was highlighted by this study.

According to the study, 46.4% of patients with severe infections exhibited mixed patterns of lung involvement (chi-square test, p < 0.05). Patients with mild infections had more of alveolar, while those with moderate infections primarily had an interstitial pattern. These types of results were also noted when comparing the pattern of lung involvement with the duration of stay. It was noted that patients with alveolar involvement had a shorter stay in the hospital when compared to the interstitial and mixed pattern of involvement.

We could not analyze the lung sequelae statistically since 42 patients were lost to follow up but based on observation it was noted that most patients with alveolar lung involvement had complete resolution of lung findings along with a few from the interstitial group. The rest of the patients from interstitial and mixed pattern had residual lung fibrosis which resulted in the requirement of chest physiotherapy and some even required long-term oxygen therapy.

## Conclusions

History has recorded numerous pandemics in past years, proving that pandemics are not a one-time occurrence. In order to start therapy at the appropriate time, there is a need for ongoing research into viruses and early severity assessment. Imaging techniques have advanced significantly in this area. The quick analysis of the patient's lung involvement offered by HRCT chest allowed for the early administration of anticoagulants and steroids, potentially saving many lives. We propose that the pattern of lung involvement plays an equal or greater role in determining the severity. This was the conclusion drawn from our investigation as well. It can assist in more accurate patient triage, reducing the disease's health impact on the healthcare system.
